# Effects of a Gluten-Free Diet on Gastrointestinal Symptoms and Quality of Life in Non-celiac Gluten-Related Disorders: A Systematic Review and Meta-Analysis

**DOI:** 10.7759/cureus.109547

**Published:** 2026-05-24

**Authors:** Shabnam A Memon, Ghazala S Virk, Brahmaiahchari Rangachari, Harshni Kalimuthu, Ishani Jayantibhai Trada, Muhammad Sohail S Mirza, Ahmed Mubarak

**Affiliations:** 1 Obstetrics and Gynaecology, CU Shah Medical College, Ahmedabad, IND; 2 Internal Medicine, Avalon University School of Medicine, Youngstown, USA; 3 Biomedical Sciences, Kentucky College of Osteopathic Medicine, University of Pikeville, Pikeville, USA; 4 Medical School, The Tamil Nadu Dr. M.G.R. Medical University, Chennai, IND; 5 Internal Medicine, American University of Barbados, Bridgetown, BRB; 6 Internal Medicine, Shandong University School of Medicine, Jinan, CHN; 7 Medical School, University of Medical Sciences and Technology, Khartoum, SDN

**Keywords:** gastrointestinal symptoms, gluten-free diet, meta-analysis, non-celiac gluten-related disorders, non-celiac gluten sensitivity, non-celiac gluten sensitivity (ncgs), quality of life (qol)

## Abstract

Non-celiac gluten sensitivity (NCGS) is a gastrointestinal and extraintestinal condition characterized by symptoms triggered by the ingestion of gluten, but it does not involve celiac disease (CD) or an allergic reaction to wheat. This meta-analysis and systematic evaluation were intended to determine the impact of a gluten-free diet (GFD) on gastrointestinal symptom reduction and quality of life (QoL) in NCGS patients. We filtered studies published from 2010 to 2025, focusing on randomized controlled trials and observational studies. The findings showed that GFD significantly promoted gastrointestinal signs and symptoms and QoL, and the pooled effect was 0.88 (95% CI = 0.20-1.55), which showed a marginal effect. Nonetheless, significant heterogeneity was observed (I² = 97.77%), suggesting that variability in effects may be attributed to differences in study design, participant characteristics, and adherence to the diet. Subgroup analysis suggested a larger pooled effect among participants with NCGS (pooled effect size = 0.93) than among participants with CD or less clearly defined gluten-related sensitivity (pooled effect size = 0.74). However, high heterogeneity within the NCGS subgroup indicates the need for further well-designed studies. The evaluation indicates that adherence and baseline symptom severity are key factors influencing the efficacy of diet. Publication bias was not identified as a major concern, as suggested by the relatively symmetrical funnel plot and the non-significant results of the statistical tests. Overall, this meta-analysis suggests that a GFD may be a useful intervention for patients with NCGS; however, individualized dietary management is needed because responses may vary across patients.

## Introduction and background

The gluten-free diet (GFD) has been given great attention over the past several years, which can be mostly attributed to its effectiveness in managing celiac disease (CD) [1,2]. Nevertheless, it has been applied to patients with non-celiac gluten sensitivity (NCGS), which has the same gastrointestinal and extraintestinal manifestations as CD, but without the autoimmune signs and intestinal damage characteristic of the latter disease [3]. NCGS is a controversial and clinically challenging diagnosis because it does not have distinct biomarkers, and the pathophysiology of this disorder is not completely understood. Although lifelong adherence to a GFD is the standard treatment for CD, the long-term therapeutic role and clinical efficacy of this dietary approach in NCGS have not yet been definitively established [4].

NCGS has become popular as the popularity of gluten-free products has surged among individuals who were not diagnosed with CD or are not allergic to wheat [5]. Despite the increasing trend, scientific evidence to justify the popular application of a GFD to treat NCGS is limited, and several studies have cast doubt on the efficacy of the diet to treat symptoms in this population [6,7]. NCGS is associated with symptoms such as stomach pain, bloating, and exhaustion, which typically disappear when gluten is not present in the diet [8]. However, it is far easier to be confused with different problems, together with irritable bowel syndrome (IBS), and its diagnosis and treatment become complex [9].

Though it is useful within the treatment of CD, GFD is a tremendous task for those with NCGS [10]. The GFD, in CD patients, addresses extreme, long-term complications, which include intestinal damage and malabsorption [11]. On the other hand, with NCGS patients, the benefits of a GFD are not so clearly outlined, and the question of whether the long-term psychological and nutritional impact of such a restrictive diet outweighs its benefits remains unresolved [12]. Studies have suggested that although a subgroup of patients with NCGS reports the resolution of gastrointestinal symptoms on a GFD, there is no convincing evidence that the outcome on extraintestinal symptoms, such as fatigue and mood disorders, is impacted [13].

In addition, GFD can have an unbalanced nutritional composition, and it may be deficient in essential nutrients such as fiber, iron, and B vitamins [14]. This has prompted doubts about the health implications of a GFD over the long term when individuals without CD are consuming it, as gluten-free foods tend to be more expensive and less nutritious than gluten-containing ones. However, accumulating evidence shows that a GFD may hold some treatment advantages in individuals with NCGS. Although some studies suggest potential symptomatic benefits of a GFD in NCGS, the biological mechanisms underlying these effects remain insufficiently established [15].

There are also alternative dietary interventions that have been used to treat NCGS, such as the low FODMAP (fermentable oligosaccharides, disaccharides, monosaccharides, and polyols) diet, which concentrates on other potential triggers of symptoms of gluten-related disorders [16]. Adding polyphenols to the diet has also been proposed as a potential intervention to address gluten-related toxicity and gluten-induced inflammation, even though research on this issue is still in its early stages [10].

This systematic review and meta-analysis aim to evaluate the effect of a GFD on individuals with NCGS, focusing on clinical outcomes, potential side effects, and the underlying processes. Through an in-depth evaluation of the most recent evidence, the evaluation aims to shed light on the efficacy of a GFD in NCGS and contribute to the present debate on the benefits and drawbacks of this dietary regimen in people who do not have CD. By synthesizing one-of-a-kind studies, this overview may be useful to direct clinical exercise; however, future studies will allow the development of more effective methods of dealing with NCGS.

## Review

Methods

Data Sources and Search Strategy

The search strategy employed in this study is detailed in Table 1.

**Table 1:** Database search strategy.

Database	Search terms used	Filters used	Search syntax
PubMed	(“non-celiac gluten sensitivity” OR “non-celiac wheat sensitivity” OR NCGS OR NCWS) AND (“gluten-free diet” OR GFD) AND (“gastrointestinal symptoms” OR “quality of life” OR “symptom relief”)	Human studies that were not older than 2010 and were published in English were considered.	The use of the operators “AND” and “OR” was made, as well as the parentheses, which were used to combine related search words.
Web of Science	TS=(“non-celiac gluten sensitivity” OR “non-celiac wheat sensitivity” OR NCGS OR NCWS) AND TS=(“gluten-free diet” OR GFD) AND TS=(“gastrointestinal symptoms” OR “quality of life” OR “clinical outcomes”)	Only human studies published in the English language between 2010 and 2025 were included.	Wildcard symbols (such as *) were employed to allow capturing such plural or variant forms of keywords.
Google Scholar	“non-celiac gluten sensitivity” AND “gluten-free diet” AND “gastrointestinal symptoms” OR “quality of life”	All articles published in the English language from the year 2010 to 2025 were used.	The use of the Boolean operators “AND” and “OR” was done. Precise phrases were put in quotation marks and wildcards like *, where necessary.

Inclusion and Exclusion Criteria

The PICOS (population, intervention, comparison, outcomes, and study design) framework was used for the study selection process, with inclusion and exclusion criteria carefully established to identify the studies relevant to the research objectives of this meta-analysis (Table 2).

**Table 2:** PICOS framework employed in the study. PICOS: population, intervention, comparison, outcomes, and study design; NCGS: non-celiac gluten sensitivity; GFD: gluten-free diet; QoL: quality of life.

PICOS element	Inclusion criteria	Exclusion criteria
Population	Individuals diagnosed with NCGS were identified based on clinical manifestations of the disorder in the presence of celiac disease, and those with an allergy to wheat were excluded	Individuals diagnosed with celiac disease or a wheat allergy
Intervention	Research investigating the impact of a GFD on symptoms related to NCGS, in which GFD is the major dietary intervention	Research that evaluated other dietary methods with no particular emphasis on a GFD
Comparison	Eligible studies included randomized controlled trials, observational studies, and single-arm pre-post intervention studies that evaluated outcomes before and after a GFD intervention	Studies with a group of comparisons, e.g., individuals on a standard diet or a placebo
Outcomes	Primary outcomes were gastrointestinal symptoms, extraintestinal symptoms, or changes in inflammatory biomarkers. The secondary outcomes were QoL, improvement, or overall health status	Clinical outcomes not concerning NCGS symptoms or QoL
Study design	Randomized, controlled, cohort, and observational studies	Case reporting, animal or laboratory investigations, narrative analyses, and investigations with insufficient methodology

Data Extraction

In this systematic review, data extraction was conducted using a standard form to ensure that it was achieved in a consistent and correct way by two reviewers. We included randomized controlled trials (RCTs), cohort studies, and observational studies that met the inclusion criteria. We extracted the sample size, age, gender distribution, and comorbidities that might affect the results of NCGS. In particular, the manner in which studies defined NCGS and diagnostic criteria was recorded. GFD data were recorded along with the type of weight loss plan applied, the period of the intervention, and any compliance check or adherence stage, as outlined by the studies. Research that studied the results of pure GFD or combined interventions was categorized. Gastrointestinal (e.g., abdominal pain and bloating), extraintestinal (e.g., fatigue and mood disturbances), and quality of life (QoL) outcomes were the main outcomes of interest. Reported secondary outcomes, including biomarkers of inflammation or clinical assessment changes, were also noted. Where differences between the two reviewers occurred, they negotiated on the findings. In case of any disagreements, the information was submitted to a third reviewer for a final decision to ensure that the data extraction process was applied uniformly and correctly to all the included studies.

Quality Assessment

The quality and risk of bias of the studies were evaluated with the help of corresponding tools based on the study design. In the case of RCTs, the Cochrane Risk of Bias 2 (RoB 2) tool was used [17]. The tool includes five areas that are key to the core validity of RCTs, which include the randomization process, allocation concealment, participant and outcome assessors’ blinding, missing data handling, and selective reporting of outcomes. The domains were evaluated as having a low, high, or unclear risk of bias in the information presented in the studies [17]. For observational studies and cohort studies, the Newcastle-Ottawa Scale (NOS) was used [18]. This scale is used to evaluate the quality of non-randomized studies in three essential domains, i.e., the selection of study groups, comparability of study groups, and outcomes assessment. Studies received up to nine stars, with more points suggesting reduced risk of bias and improved methodological quality [18].

Along with the quality of each observation, publication bias was evaluated by way of visual inspection of funnel plots, asymmetry of which may highlight selective reporting or a small study contributing a disproportionately large effect. To further investigate the potential of publication bias, a statistical test of asymmetry of funnel plots, i.e., Egger regression, was conducted. The trim-and-fill method was used to correct the results in the case of high publication bias to obtain a more precise and less biased estimate of the overall effect size [19].

Statistical Analysis

A random-effects model was used to pool the data due to the anticipated dissimilarity in research features, population, intervention, and effects. The random-effects model was chosen, as it takes into consideration the variability of the studies and provides a more trustworthy and generalizable result if the studies are not equal in terms of design or the features of the participants. The standards of the comparison included standardized mean differences (SMDs) with 95% confidence intervals (CIs) to evaluate the accuracy of the estimates of the continuous outcomes (e.g., improvement in gastrointestinal symptoms, such as abdominal pain and bloating, and extraintestinal symptoms, such as fatigue and mood disturbances). In case of dichotomous outcomes, odds ratios (ORs) were estimated to identify the likelihood of improvement in the GFD group relative to that of the control group.

I² was used to ascertain the level of heterogeneity of the included studies. The statistic measures the proportion of the overall variation between studies that is not because of probability alone. The I² values were categorized as low heterogeneity (0-25%), moderate, and high heterogeneity (50-100%). The excessive heterogeneity meant a large variation in the treatment outcomes throughout the studies, which necessitated additional research. Subgroup analyses were performed to determine the reasons for heterogeneity. These were conducted on such criteria because they take a look at layout (e.g., RCTs vs. observational studies), compliance with the GFD (e.g., strict adherence vs. self-mentioned adherence), characteristics of the participants (e.g., age, gender, and comorbidities), and period of the intervention (e.g., short-term vs. long-term gluten-free food regimen).

Results

Study Selection

**Figure 1:**
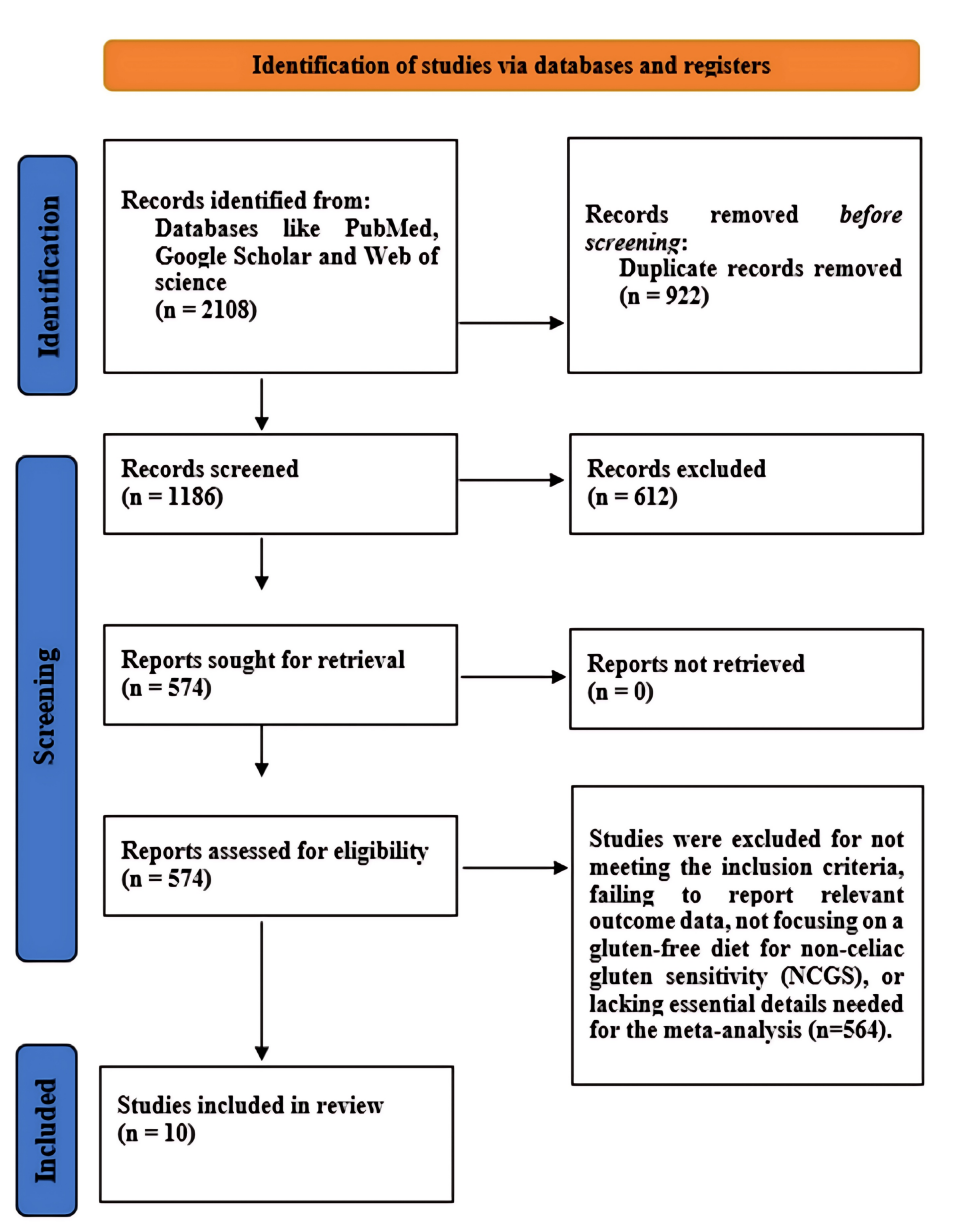
Preferred Reporting Items for Systematic Reviews and Meta-Analyses (PRISMA) flowchart.

An initial search across databases, including PubMed, Google Scholar, and Web of Science, yielded 2,108 studies. After removing 922 duplicate records, 1,186 studies proceeded to title and abstract screening. During this phase, 612 studies were excluded as they did not address gastrointestinal symptoms or QoL in the context of NCGS. Consequently, 574 full-text articles were retrieved and evaluated for eligibility. Upon thorough full-text review, 564 articles were excluded, primarily due to the absence of GFD interventions in NCGS patients, lack of pertinent outcome data, or insufficient methodological details necessary for inclusion in the meta-analysis. Ultimately, 10 studies met the predefined criteria and were incorporated into the systematic review and meta-analysis to assess the impact of a GFD on gastrointestinal symptoms and QoL among individuals with NCGS (Figure 1) [20].

Study Characteristics

The studies included in this systematic review and meta-analysis mostly investigated the effects of a GFD in patients with NCGS and some comparisons to other disorders (e.g., IBS, fibromyalgia (FM), and CD) (Table 3). Most studies had a prospective design while using a pre/post-intervention approach, enabling within-subject comparisons to determine the impacts of GFD on gastrointestinal (QoL) and immune responses. Key outcomes across studies included gastrointestinal symptoms (e.g., bloating and abdominal pain), extraintestinal symptoms (e.g., fatigue and mood disorders), intestinal motility, gut microbiota composition, and immune markers (e.g., cytokines and anti-gliadin antibodies). Other studies also involved gluten challenges, a comparison between GFD and gluten consumption, to demonstrate the existence of NCGS. Moreover, some studies combined low FODMAP diets and GFD to investigate the interaction. The majority of the studies showed that symptoms improved significantly, with gastrointestinal discomfort and QoL improving after GFD. However, some studies also suggested that some patients experience persistent symptoms. Most studies confirmed the positive impact of GFD on the gastrointestinal and immune systems.

**Table 3:** Summary of studies included in the review. NCGS: non-celiac gluten sensitivity; NCWS: non-celiac wheat sensitivity; FM: fibromyalgia; GFD: gluten-free diet; CD: celiac disease; QoL: quality of life; IBS: irritable bowel syndrome; FODMAP: fermentable oligosaccharides, disaccharides, monosaccharides, and polyols; IBS-QoL: Irritable Bowel Syndrome Quality of Life questionnaire; IBS-SSS: Irritable Bowel Syndrome Severity Scoring System; EQ-5D: EuroQol 5-Dimension questionnaire; CDQ: Celiac Disease Questionnaire.

Study	Study design	Population	Intervention	Comparison	Outcomes	Key findings
Caio et al. [21]	Prospective observational study with a pre-post design	Patients with NCGS, diagnosed based on clinical symptoms, and negative for CD and wheat allergy	GFD for 6 months, clinical and immunological evaluation pre- and post-GFD	Pre-GFD vs. post-GFD within the same NCGS patients: comparison with CD patients	Anti-gliadin antibodies (IgG and IgA) and clinical response (gastrointestinal and extraintestinal symptoms)	Significant reduction in anti-gliadin IgG levels after GFD in NCGS patients, correlating with strict adherence and symptom improvement; IgA levels also decreased in good responders to GFD
Elli et al. [22]	Randomized, double-blind, placebo-controlled gluten challenge with a pre-post design	Patients with functional gastrointestinal symptoms (IBS, etc.), suspected of having NCGS	GFD followed by a double-blind, placebo-controlled gluten challenge	Pre-GFD vs. post-GFD within the same patients: comparison of gluten vs. placebo challenge	Gastrointestinal symptoms, QoL, and general well-being	Significant improvement in symptoms occurred during the GFD phase; symptoms worsened during the gluten challenge, confirming NCGS diagnosis in responsive patients
Barmeyer et al. [23]	Prospective observational study with a pre-post design	Patients with diarrhea-dominant and mixed-type IBS, diagnosed with NCWS	Long-term GFD (12 weeks to 24 months); clinical evaluation pre- and post-GFD	Pre-GFD vs. post-GFD within the same patients	Gastrointestinal symptoms (pain, bloating, diarrhea), QoL, symptom relief (IBS-QoL, IBS-SSS, EQ-5D)	Significant improvement in gastrointestinal symptoms and QoL; long-term adherence to GFD showed symptom relief and better QoL in IBS patients with NCWS
Haro et al. [24]	Prospective intervention with two dietary phases (baseline GFD vs. low-gliadin bread)	Patients with NCGS	Consumption of transgenic low-gliadin wheat bread (E82) vs. gluten-free bread (standard GFD)	Within-subject comparison of two dietary phases (basal GFD vs. low-gliadin bread)	Gastrointestinal symptoms, gut microbiota composition (via sequencing and stool samples)	No significant differences in gastrointestinal symptoms between GFD and low-gliadin bread; better gut microbiota profile with low-gliadin bread compared to GFD
Dieterich et al. [25]	Prospective sequential diet intervention with pre-post measurements	Patients diagnosed with NCGS and healthy controls	Low FODMAP diet followed by GFD; compliance monitored and diet phases compared sequentially	Pre- vs. post-diet intervention within NCGS patients; comparisons with healthy controls	Gastrointestinal symptoms, psychological symptoms, intestinal inflammation (intraepithelial lymphocytes and goblet cells), gut microbiota composition	Both low FODMAP and GFD improved gastrointestinal symptoms. GFD additionally modulated gut microbiota composition, and histological improvements were observed in intestinal samples
Roncoroni et al. [26]	Prospective diet intervention with pre-post measurements	Patients with NCGS are diagnosed based on clinical symptoms and exclusion of CD and wheat allergy	GFD for 3 weeks, followed by the reintroduction of gluten (low, mid, high)	Pre-GFD vs. post-GFD within the same patients: comparison of different gluten doses (low, mid, high)	Gastrointestinal symptoms, QoL, and general well-being after gluten reintroduction	Symptoms worsened with higher gluten doses, with significant symptom improvement after GFD. A moderate reintroduction dose showed some symptom relief, but not to the same degree
Tovoli et al. [27]	Prospective data collection with retrospective analysis; single-arm follow-up study of NCWS patients	Adults with NCWS, diagnosed by the Salerno criteria	GFD for at least 1 year; compliance monitored; patients with low adherence excluded	Pre- and post-GFD within the NCWS group; the CD group included for QoL comparison	Gastrointestinal and extraintestinal symptom prevalence and severity, QoL measured by the CDQ	Significant reduction in symptom prevalence and severity after GFD; QoL improved, but persistent symptoms remained in some NCWS patients
Moleski et al. [28]	Randomized, double-blind, placebo-controlled crossover trial	Patients with NCGS, including healthy controls for comparison	GFD followed by gluten challenge with two doses (low and high)	Gluten vs. placebo challenge in NCGS patients and healthy controls	Gastrointestinal symptoms (bloating, pain, diarrhea), Celiac Symptom Index for symptom severity	Gluten ingestion triggered gastrointestinal symptoms in NCGS patients, with severity correlating to gluten dose; placebo challenge had no significant effect
Schinocca et al. [29]	Prospective intervention with a pre-post design	Patients with FM and NCWS	GFD for 8 weeks; clinical evaluation pre- and post-GFD	Pre-GFD vs. post-GFD within the same patients	Gastrointestinal symptoms, QoL, immune response (cytokines, T-cell production)	Significant reduction in FM-related symptoms (pain, fatigue) and gastrointestinal symptoms post-GFD; modulation of immune response (decrease in pro-inflammatory cytokines, increase in IL-5)
Cobos-Quevedo et al. [30]	Prospective observational study with a pre-post design	Individuals with CD and NCGS	GFD for 4 weeks; adherence monitored with food diaries and weekly check-ups	Pre-GFD vs. post-GFD in CD and NCGS patients: no separate control group	Gastrointestinal motility (gastric, small bowel, colonic transit times), contractility, and pH levels	Improved motility in the small intestine and colon in CD patients; NCGS patients showed improved intestinal motility, but no effect on colonic transit

Quality Assessment

**Figure 2:**
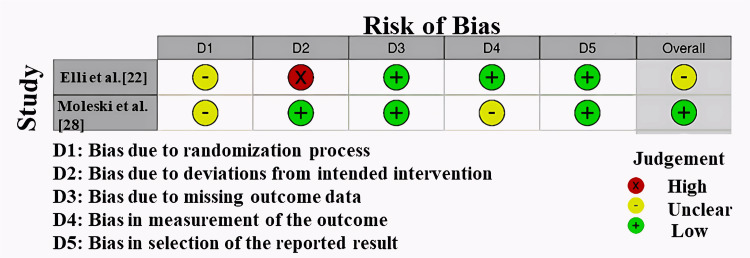
Bias assessment using the Risk of Bias 2.

Risk of bias: The risk of bias assessment for the studies included in this meta-analysis is presented in Figure 2. The risk of bias assessment indicated an overall low risk of bias across most evaluated domains for both Elli et al. [22] and Moleski et al. [28]. Elli et al. [22] had unclear concerns in allocation concealment (D2), indicating some uncertainty about how individuals were assigned to groups, which can introduce capacity bias. However, other domains, such as randomization, blinding, and outcome assessment, were rated as low risk. In contrast, Moleski et al. [28] demonstrated low risk across all domains, suggesting strong methodological rigor. Overall, both studies were considered to have reliable findings, with Elli et al. [22] showing a slight uncertainty in one aspect but still maintaining overall credibility [31].

**Figure 3:**
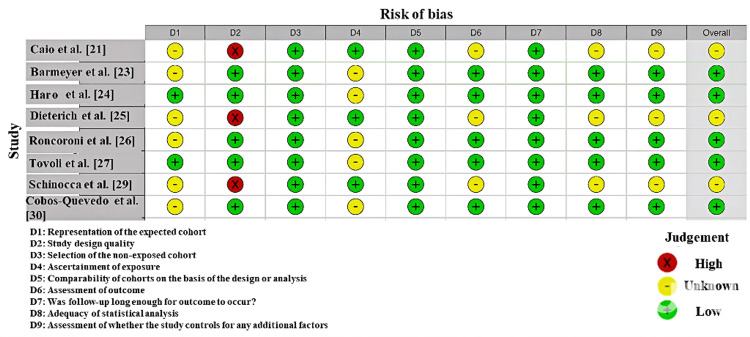
Bias assessment using the Newcastle-Ottawa Scale.

The studies by Haro et al. [24] and Dieterich et al. [25] were observed to have a low risk of bias in all domains, indicating good methodological rigor with adequate controls in dealing with confounding factors. The unclear risks of Tovoli et al. [27] and Cobos-Quevedo et al. [30] suggest inadequate reporting on some of the domains, making it difficult to appropriately quantify the bias in these studies. In general, most studies had a low risk of bias, which increased the validity of the pooled results. Nevertheless, the high-risk domains in certain studies, especially in the selection of participants and measurement of outcomes, should be considered when interpreting the results because they might lead to the observed effects (Figure 3) [32].

**Figure 4:**
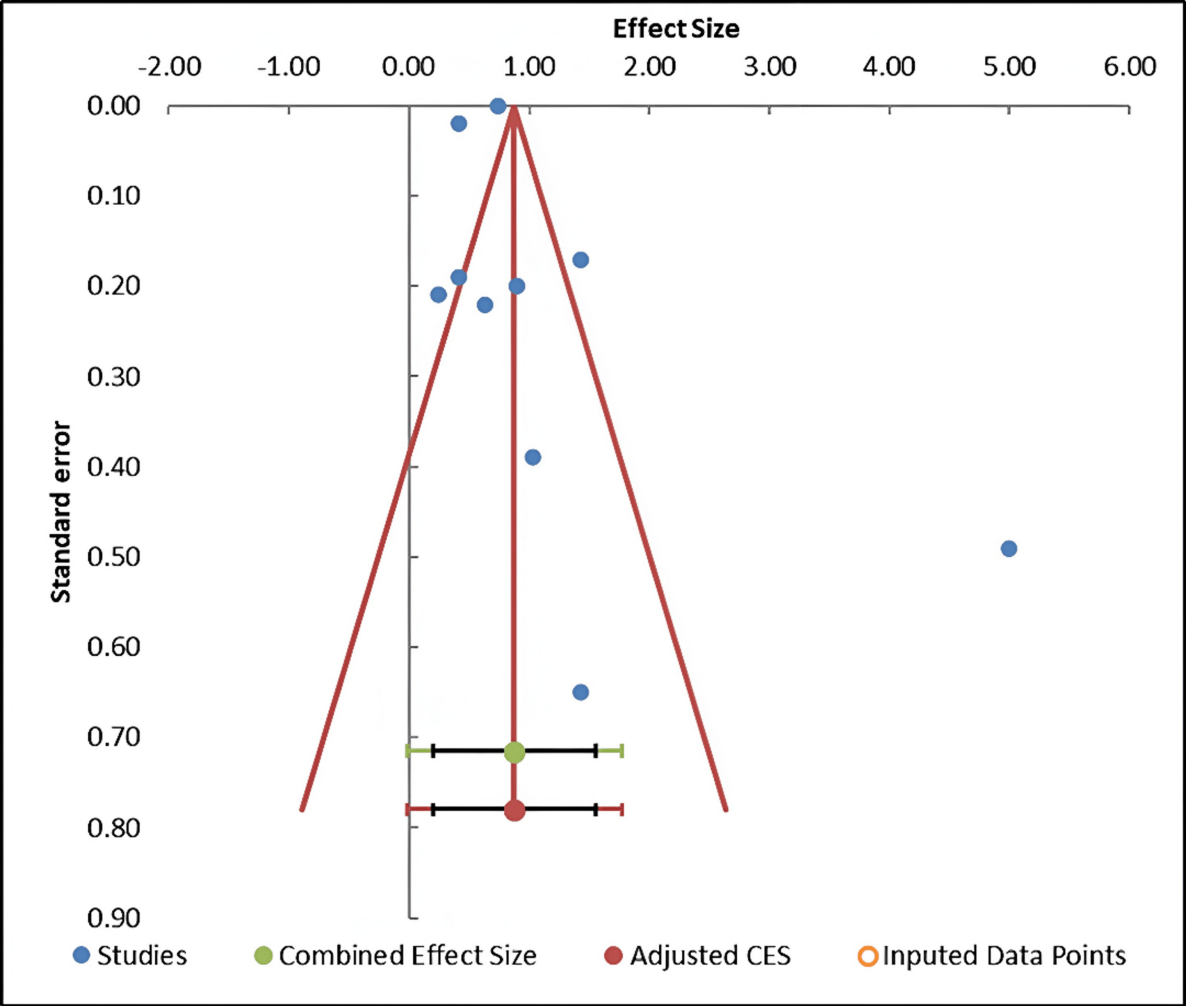
Funnel plot measuring publication bias in the studies. CES: combined effect size.

Publication bias: The funnel plot indicated that the studies were fairly evenly distributed around the overall effect, indicating that publication bias was unlikely to be a significant issue in this meta-analysis. The studies were also well distributed above and below the combined effect size, with smaller sizes appearing at the bottom and larger sizes at the top, which is common in a balanced funnel plot (Figure 4). This symmetry means that both small and large studies were covered, and there was no selective publication of positive results. This finding is also reinforced by the Egger analysis of regression (Table 4). The slope was 53.92 with a p-value of 0.074, which is not less than the significance level of 0.05. Hence, there was no statistically significant evidence of asymmetry in the funnel plot, which supports the conclusion that there is no strong evidence of publication bias in this analysis (Table 5). Finally, the trim and fill analysis, which identifies missing studies to offset any asymmetry in the funnel plot, indicated that there were no missing studies that needed to be imputed. Hence, the studies were evenly spread in the analysis process, and there was no significant publication bias [33].

**Table 4:** Egger regression. SE: standard error; CI: confidence interval; LL: lower level; UL: upper level.

	Estimate	SE	CI LL	CI UL
Intercept	5.92	2.88	-0.59	12.44
Slope	-1.07	0.98	-3.30	1.15
t test	2.06	Not applicable	Not applicable	Not applicable
P-value	0.074	Not applicable	Not applicable	Not applicable

**Table 5:** Funnel plot findings. SE: standard error; CI: confidence interval; PI: prediction interval; LL: lower level; UL: upper level.

Study name	Effect size	SE (z)
Caio et al. [21]	5.00	0.49
Elli et al. [22]	0.74	0.22
Barmeyer et al. [23]	0.41	0.02
Haro et al. [24]	0.25	0.21
Dieterich et al. [25]	1.43	0.65
Roncoroni et al. [26]	0.90	0.20
Tovoli et al. [27]	1.03	0.39
Moleski et al. [28]	1.43	0.17
Schinocca et al. [29]	0.41	0.19
Cobos-Quevedo et al. [30]	0.63	0.22
Combined effect size	NA	NA
Combined effect size	Observed	NA
Effect size	0.88	NA
SE (z)	0.30	NA
CI LL	0.20	NA
CI UL	1.55	NA
PI LL	-0.02	NA
PI UL	1.77	NA
Heterogeneity	NA	NA
Q	403.97	NA
p_Q_	0.000	NA
I^2^	97.77%	NA
Z	0.07	NA
T	0.26	NA

**Figure 5:**
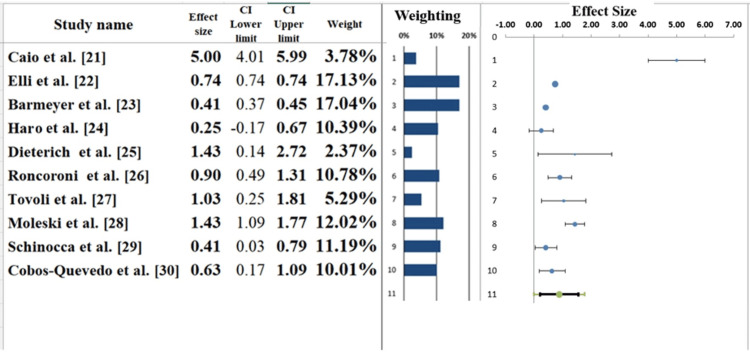
Forest plot exemplifying effect size estimates from each study.

Forest plot: The forest plot in Figure 5 presents the meta-analysis findings of various studies on the impact of GFD on different health outcomes, namely, in patients with NCGS. The pooled effect size of 0.88 (95% CI = 0.20-1.55) was obtained in a random-effects model. This shows that the GFD has a moderate overall impact on improving symptoms in NCGS patients; however, the CI highlights that the actual impact may be a small positive effect or a null effect. Individual studies had a diverse contribution to the overall effect. Caio et al. [21] had a significantly large effect size of 5.00 (CI = 4.01-5.99), with a strong positive response to the GFD, and Moleski et al. [28] had a much smaller effect size of 1.43 (CI = 1.09-1.77), which was much more moderate and consistent. On the other hand, Schinocca et al. [29] suggested that the effect size was not very large; therefore, the diet may not have much influence on a certain population, or there may be a variation in the findings of other studies. The length of the horizontal bars represents the weight of each study in the pooled effect size, with studies such as Elli et al. [22] and Roncoroni et al. [26] having more weight because they had more participants. These studies indicate the increased level of impact on the conclusion in general [34].

Heterogeneity assessment: According to the forest plot and Table 6, substantial heterogeneity was observed among the included studies. The I² value was 97.77%, indicating considerable between-study variability rather than moderate heterogeneity. The Q-statistic was 403.97 with a p-value <0.001, showing that the variation in effect sizes was statistically significant and unlikely to be due to chance alone. This heterogeneity may be explained by differences in study populations, intervention types, outcome measures, dietary adherence, and study designs. The between-study variance was T² = 0.07, as reported in Table 6. Therefore, the pooled estimate should be interpreted cautiously and considered alongside the subgroup analysis and narrative synthesis [35].

**Table 6:** Information correlating with the forest plot. SE: standard error; CI: confidence interval; PI: prediction interval; LL: lower level; UL: upper level.

Meta-analysis model	
Effect size	0.88
SE	0.30
CI LL	0.20
CI UL	1.55
PI LL	-0.02
PI UL	1.77
Z-value	2.95
One-tailed p-value	0.002
Two-tailed p-value	0.003
Number of included studies	10
Heterogeneity	
Q	403.97
p_Q_	0.000
I^2^	97.77%
T^2 ^(z)	0.07
T (z)	0.26

Subgroup Analysis

**Figure 6:**
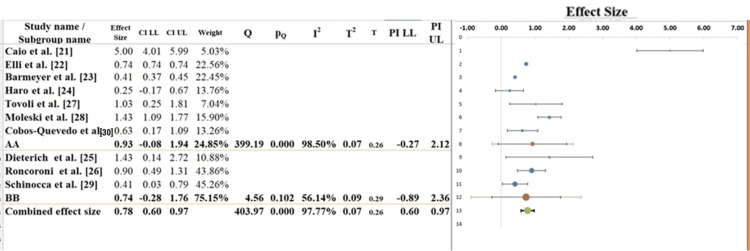
Subgroup analysis of included studies investigating the effects of GFD on gastrointestinal symptoms and QoL in patients with NCGS, depending on study characteristics such as patient population (NCGS vs. celiac disease) and diet adherence. NCGS: non-celiac gluten sensitivity; GFD: gluten-free diet; QoL: quality of life; CI: confidence interval; PI: prediction interval; LL: lower level; UL: upper level.

Subgroup analysis (Figure 6) categorized the studies into two groups, Group AA and Group BB, according to the major study characteristics, including patient population or intervention type. This difference is important because it emphasizes how some factors, including patient characteristics or the nature of the intervention, could affect the overall effect size. Group AA included research discussing the impact of a GFD on NCGS patients. The effect size of Group AA was 0.93 (95% CI = -0.08 to 1.94), which indicated a moderate intervention effect. The heterogeneity of this subgroup was also large, with an I² of 98.50, indicating that the results of the studies varied significantly. The heterogeneity was statistically significant, as can be seen from the Q-statistic of 399.19 (p = 0.000). Such variability can be explained by variations in the population of the studies, measurement instruments, or adherence to the GFD. The prediction interval (PI) for Group AA was -0.27 to 2.12, indicating that in a similar future study, the true effect size may vary significantly (Table 7).

**Table 7:** Information regarding subgroup analysis. SE: standard error; CI: confidence interval; PI: prediction interval; LL: lower level; UL: upper level.

Meta-analysis model	
Effect size	0.78
SE	0.08
CI LL	0.60
CI UL	0.97
PI LL	0.60
PI UL	0.97
Number of included subjects	691
Number of subgroups	2
Analysis of variance	N/A
Between/Model (Q*)	0.52
Between/Model (df)	1
Between/Model (P)	0.470
Within/Residual (Q*)	67.51
Within/Residual (df)	8
Within/Residual (P)	0.000
Total (Q*)	68.03
Total (df)	9
Total (P)	0.000
Pseudo R^2^	0.77%

Group BB included studies focusing on the effects of the GFD in patients with CD or those suffering from less severe forms of gluten sensitivity. The pooled effect size for Group BB was 0.74 (95% CI = -0.28 to 1.76), which was smaller and less consistent than the effect for Group AA. The heterogeneity for Group BB was moderate, with an I² of 56.14% and a Q-statistic of 4.56 (p = 0.102), indicating that the variability in the study results was less pronounced than for Group AA, although it was still noticeable. The range of the prediction of Group BB was -0.89 to 2.36, implying that the possible results were more varied and likely included the possibility of no effect. The test for subgroup differences (p = 0.000) indicated that the difference between the effect sizes of Group AA and Group BB was statistically significant. This implies that the effect of the intervention was likely to differ according to the type of population (e.g., NCGS patients vs. CD patients) and that there could be variables such as severity of gluten sensitivity, adherence to GFD, or other unmeasured variables that may account for the differences in effect sizes [36].

Narrative Analysis

This meta-analysis reviewed the studies on the impact of GFD on those with NCGS, both within the short- and long-term symptom remission. The studies had several designs as they included RCTs, potential observational studies, and eating regimen interventions, which enabled researchers to have a full picture of the results of GFD in diverse groups of patients, a range of symptoms, and the routes of the intervention. These disparities allowed the appearance of a subtle image of the effect of GFD on gastrointestinal signs and symptoms and QoL.

Gastrointestinal Symptom Relief

Most studies showed that the gastrointestinal signs and symptoms, together with stomach pain, bloating, and diarrhea, improved substantially while on a GFD. Evidence, collectively with Caio et al. [21] and Moleski et al. [28], confirmed excessive symptom reduction among patients with NCGS, mainly in those who had a high adherence to the weight loss plan. Nonetheless, other reviews, e.g., Roncoroni et al. [26], observed fewer full-sized effects, which might be attributed to the decreased adherence or the high levels of gluten sensitivity within individuals. These differences emphasize the role of personal factors, including the severity of symptoms within the baseline and adherence to the GFD, in successful treatment.

Adherence and Duration of the Diet

The benefit of GFD is carefully related to compliance and time. Long-term effects of the diet are greater, as stated and sustained, as visible in research that concentrated on adherence over the long-term [29]. Conversely, fewer benefits were noted in short-term interventions wherein adherence was not always steady. These consequences spotlight the importance of the affected male and female compliance with the diet plan to achieve maximum benefits.

Improvement in Quality of Life

Several studies also evaluated the QoL of patients who had NCGS before and after following a GFD. Tovoli et al. [27] and Schinocca et al. [29] also found significant improvement in QoL, as patients had improved levels of mental well-being, physical health, and energy levels. These results indicate that the dietary intervention should not only relieve gastrointestinal symptoms but should also be considered as part of the psychosocial outcome, including a better mood and functional capacity. Nevertheless, the gains in QoL were smaller in the study conducted by Barmeyer et al. [23], which suggests some variability in the processes by which different people react to GFD.

Variability Across Studies

The inconsistency in the findings of the studies can be explained by their differences in study population, compliance with diet, duration of intervention, and methodology. In some studies, a short-term intervention was provided, whereas in others, there was an evaluation of the long-term effects, leading to differences in the level of symptom improvement. The method of evaluating gastrointestinal symptoms and QoL was different, which led to discrepant results. Despite these variations, the general trend indicates that GFD is a useful intervention in symptom management of NCGS patients, especially when the diet is adhered to over longer durations.

Discussion

The systematic review investigated the impact of a GFD on individuals with a NCGS with regard to gastrointestinal symptoms and QoL. We found that gastrointestinal symptoms, including bloating, abdominal pain, and diarrhea, improved significantly despite the available literature. For instance, Fasano et al. [37] found that a GFD helped relieve symptoms in individuals with NCGS, which provides evidence regarding the involvement of gluten in gastrointestinal pain. Likewise, Caio et al. [21] reported symptom relief and found that 90% of NCGS patients had improved with the GFD, which is in line with the statistics in our meta-analysis.

Moreover, QoL was positively impacted in a few studies in this review, specifically in intellectual fitness, bodily fitness, and preferred energy. These findings are comparable with those reported by Tovoli et al. [27], who also found that NCGS patients improved their QoL significantly after a GFD, especially with fatigue reduction and emotional well-being. The improvement, however, ranged, and some studies, such as those by Barmeyer et al. [23], showed only mild improvements, particularly in cases when the diet was not followed strictly.

The difference in the treatment outcomes between studies can be explained by the variability in the study design, the level of the initial symptom severity, and dietary compliance. Elli et al. [22] found that a group of patients with increased adherence achieved significant symptom reduction, suggesting that increased adherence time and consistency may increase symptom reduction. This observation is in line with Gulati [38], who indicated that NCGS patients who follow a GFD directly correlate with symptom improvement.

The observed heterogeneity of the studies can be attributed to the variance in gluten sensitivity individually, as discussed by Cascella et al. [39]. They emphasized that the intensity of the base symptoms is very critical to the responsiveness of patients to nutritional changes. This variability was also verified by our outcomes, as patients described diverse degrees of relief based on the severity of their initial signs and comorbidities.

Limitations

Although this systematic review offers a few beneficial ideas concerning the effect of a GFD on NCGS, some limitations need to be considered. First, the heterogeneity of the research included in this meta-evaluation poses a big challenge. The varied study types, together with RCTs and observational studies, the sample sizes, and processes of comparing signs and QoL, likely impacted the uniformity of the effects. Moreover, intervention duration and adherence to the GFD had an extensive range, resulting in variations in results between studies. This inconsistency renders it hard to conclude the long-term effects of the food plan. The second limitation is that only a small number of studies directly covered NCGS, and many studies also included other disorders, such as IBS or CD. This overlap has the possibility of affecting the results, as patients with other gastrointestinal disorders might not react to a GFD. In addition, the absence of blinding in certain observational studies and the imprecise measurement of outcomes also restrict the reliability and generalizability of outcomes. Lastly, the incomplete reporting of some of the studies on dietary adherence and baseline characteristics can lead to bias, which may impact the strength of the results.

Future research

To fill in the gaps in the literature, future studies in the area of NCGS and the impact of a GFD need to concentrate on several areas. First, long-term and large-scale RCTs are required to gain a better insight into the long-term effects of GFD on gastrointestinal symptoms and QoL in NCGS patients. Although this systematic review has indicated that GFD has positive impacts, most of the studies were short-term, and long-term studies are required to establish the longevity of the effects of GFD. Second, the effect of adhering to the GFD in producing the best results should be studied in future research because the results of this review were inconsistent depending on compliance. Adherence research and its effects on symptom relief may be useful to determine more effective interventions to support long-term diet adherence. The identification of biomarkers for NCGS that could be used to diagnose and monitor patients is another promising future research area. Introduction of biomarkers would facilitate patient stratification according to the state of their condition and precisely introduce dietary interventions. Lastly, studies must be conducted to identify the psychosocial advantages of GFD, mainly its impact on mental health and widespread well-being, as our evaluation recognized significant development in these areas. Further elaboration of this factor might also help in the introduction of a more comprehensive idea of the advantages of GFD among NCGS patients.

## Conclusions

This meta-analysis and systematic review provide evidence that a GFD can often reduce gastrointestinal signs and symptoms and improve the QoL of those with NCGS. GFD led to consistent improvements in the symptoms of stomach pain, bloating, diarrhea, cognitive fitness, bodily fitness, and primary energy levels. These findings confirm the effectiveness of GFD within the management of NCGS and suggest more than gastrointestinal symptom control. Nevertheless, there was large heterogeneity in some studies, due to variation in study design, intervention length, adherence, and baseline health circumstances of the participants. Studies that had longer intervention times and better compliance confirmed stronger symptom improvement, making patient compliance a vital factor. This inconsistency implies that patients with NCGS require a customized treatment plan for optimal management. Although the general results were promising, further research is required, especially longitudinal studies, to gain insights into the sustainability of GFD results. Further research is needed regarding biomarkers and adherence techniques to enhance the treatment outcomes, as well as to improve nutritional interventions in NCGS. Altogether, this review highlights the clinical significance of GFD in the treatment of NCGS and additionally provides avenues for future research.
